# Most Personal Exposure to House Dust Mite Aeroallergen Occurs during the Day

**DOI:** 10.1371/journal.pone.0069900

**Published:** 2013-07-24

**Authors:** Euan R. Tovey, Christiana M. Willenborg, Daniele A. Crisafulli, Janet Rimmer, Guy B. Marks

**Affiliations:** 1 Allergen Group, Woolcock Institute of Medical Research, Sydney, New South Wales, Australia; 2 Sydney Medical School, the University of Sydney, Sydney, New South Wales, Australia; 3 Virology Research Laboratory, Prince of Wales Hospital, Sydney, New South Wales, Australia; 4 Epidemiology Group, Woolcock Institute of Medical Research, Sydney, New South Wales, Australia; The Ohio State University, United States of America

## Abstract

**Background:**

The bed is commonly regarded as the main site of house dust mite exposure; however this has not been directly established by continuous measurements. The objective of this study was to determine the pattern of personal exposure to mite aeroallergen over 24 hours.

**Methods:**

12 adults each collected 9 sequential samples (8 during the day, mean 115 mins, and one overnight, mean 514 mins) over 24 hours using a portable air-pump (2L/min) connected to an IOM filter located on the shoulder during the day and on the bed head overnight. Samples were analysed for mite allergen Der p 1 by ELISA. Location and activity were recorded. A mixed model analysis was performed to determine exposure as a function of 14 categories of activity.

**Results:**

Personal aeroallergen exposure differed widely over time, both within and between subjects. The highest average exposure (1117 pg/m^3^, 95% CI: 289-4314) occurred on public transport and the lowest overnight in bed (45 pg/m^3^, 95% CI: 17-17), which contributed only 9.8% (95% CI: 4.4%-15.1%) of total daily exposure. Aeroallergens were not related to bed reservoirs.

**Conclusion:**

The study challenges the current paradigm that the bed is the main site of HDM exposure and instead suggests most exposure occurs in association with domestic activity and proximity to other people. Effective mite interventions, designed to improve asthma outcomes, need to first identify and then address the multiple sources of aeroallergen exposure.

## Introduction

House Dust Mite (HDM) allergy is a significant risk factor for asthma in many countries and high exposure to the allergens contributes to airway inflammation [1] and asthma exacerbations [2]. The bed has long been regarded as the main site of this exposure [3], although direct evidence of this is lacking. The presumption about the dominance of beds is based on the high HDM allergen concentrations in bed dust compared to other sites in houses, the proportion of life spent in bed and the physical proximity between the bedding and the occupant [3]. Indirect support also came from models based on intermittent serial measurements of acute exposures [4] or from air samples collected under different conditions [5,6]. However recent data suggests HDM aeroallergen exposure in bedrooms may be low compared to those in schools and lounge rooms [7], and other studies show the pattern and intensity of personal activity is a critical factor in generating bioaerosol exposure [8]. This study aimed to model the pattern of HDM aeroallergen exposure throughout the day and night and determine its relationship to people’s activities.

## Materials and Methods

### Population

Twelve healthy adults (75% female; mean age 40.3 years, +/- SD 15.8) were recruited. Sampling occurred between January and March (late summer) in Sydney, Australia. Four subjects collected samples at home, eight at work during the day, six of whom worked in different parts of the same building. Subjects had not washed their bedlinen in the previous week and did not use bed encasings.

### Ethics statement

All subjects provided written informed consent and retained a copy of the Participant Information Sheet. The study was approved by the University of Sydney, Human Research Ethics Committee, approval number 11392.

### Sampling and assays

Subjects collected eight sequential samples, (mean 116 min +/- 68.0SD) between 7 am and 10:30 pm and a ninth sample overnight (mean 512.3 min +/- 8.3SD) using a air pump (Casella TUFF™, Bedford, UK, 2L/min), connected to an IOM (Institute of Occupational Medicine, SKC Inc. PA) sampling head. During the day the pump was carried in a back pack and the sampler was located on its shoulder strap; at night the sampler was taped onto the head of the bed and the pump kept in a sound-proof box. Samples were collected onto filters (Technostat70+ (H&V, Airfiltration Pty Ltd, Cumbria, UK) and subjects changed these between periods of related activities. A sample of bed dust was also collected, as described [9]. HDM allergen Der p 1 was analysed by ELISA (Indoor Biotechnologies Pty Ltd, Charlottesville, VA), modified to provide a sensitivity of 9.7pg/ml [10].

### Activities

Activity in each period was recorded by subjects as unstructured diary entries and images of location were also taken using a time-lapse camera (Apple iPod, running “Time Lapse Pro”) mounted on the other shoulder of the backpack. Initially 39 activities were identified, based on a *post hoc* review of the diaries and images which were summarised into fourteen categories of similar ‘activities’, as shown in Table S1.

### Analysis of data

Exposure (pg of allergen sample/m^3^ air sampled) was log-transformed unless specified. Exposures during activities were compared using mixed model regression in which subjects were assigned random intercepts, activities were fixed effects and comparisons were expressed as ratios (the anti-log of differences). For the relative contribution of different periods to total exposure, the contribution of each period was averaged, without log transformation. For bed dust reservoir samples, the concentration of allergen per dust weight was used. For modelling, the software package SAS 9.2, SAS Institute, Cary, NC, USA was used.

## Results

**Figure 1:**
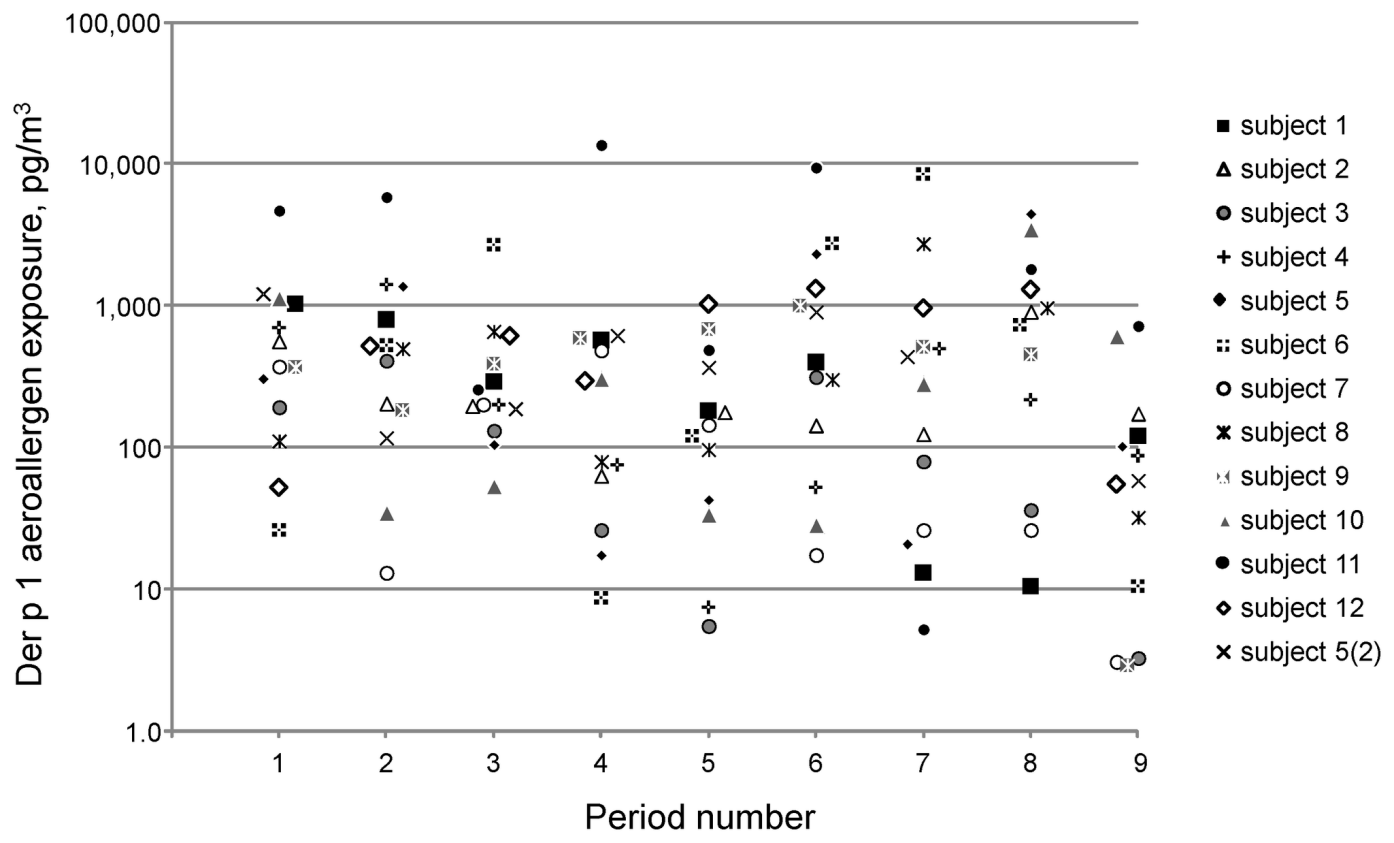
Average personal exposure (**pg/m** ^**3**^) **for the nine sequential periods over 24 hours.** Periods 1-8 occurred over the day at approximately 2 hour intervals between 7:00am and 10:30pm and Period 9 was overnight (~8 hours). Each symbol represents a separate subject. One subject (5) who collected samples during the working week and again at the weekend is shown as subject 5(2).

Aeroallergen was detected in 83.6% of the 116 IOM samples (one person provided 8 additional samples on a second day of sampling). Plots of exposure (pg /m^3^) for each of the nine sequential periods are shown in Figure 1; each symbol represents one subject. Figure S1 shows the same data as Figure 1, but re-organised by person to demonstrate exposures in the different periods. Table 1 shows information for each of the nine sequential periods on sampling times, geometric mean quantity of allergen, the exposure (pg/m^3^), the proportion of total exposure and the number of different activities. Period 9, “in bed, overnight” on average contributed 9.8% (95% CI = 4.4-15.1) of the total quantity of aeroallergen exposure.

The exposures associated with the14 activities, as determined by mixed model analysis of the nine samples for each subject, are shown in Table 2. Most exposure occurred when the subjects were active in either domestic or crowded public situations. Only two activities were significantly different than the mean of the other exposures; ‘Travel, public transport’ was significantly higher, (ratio = 5.80, 95% CI: 1.50-22.48, p<0.01) and ‘In-bed overnight’ was significantly lower (ratio = 0.17, 95% CI: 0.07-0.45, p<0.001). There was no correlation between the concentration of allergen in bed reservoirs and aeroallergen either overnight (Period 9) (r^2^=0.09, P =0.3) or as cumulative exposure (Periods 1-9) (r^2^=0.20, P=0.2).

**Table 1:** Times, exposures and activities for sampling periods 1-9, as collected on 13 occasions.

Period	Sampling time, min	Aeroallergen, pg/period	Percent of the total quantity of exposure	Aeroallergen exposure pg/m^3^	Number of activities per period
	GM (+/- 95%CI)	GM (+/- 95%CI)	Mean % (+/- 95%CI)	GM (+/- 95%CI)	
1	90.7 (70.4-116.8)	68.6 (33.1-143.1)	13.6 (6.9-20.3)	379.0 (177.2-810.9)	4
2	66.7 (53.7-82.8)	46.2 (21.3-100.4)	9.1 (3.9-14.4)	346.4 (144.8-828.8)	9
3	173.2 (138.1-217.2)	92.7 (53.8-159.4)	12.6 (7.5-17.8)	267.5 (157.8-453.5	7
4	76.3 (59.3-98.1)	27.3 (11.2-66.7)	6.4 (2.2-10.7)	179.0 (62.3-514.6)	7
5	145.7 (110.4-192.4)	32.6 (16.5-64.4)	4.9 (2.8-4.1)	111.8 (46.6-267.9)	8
6	80.3 (56.4-114.5)	66.5 (22.3-198.4)	14.2 (6.8-21.6)	414.2 (150.1-1143.1)	7
7	91.8 (67.3-125.2)	32.7 (10.4-109.1)	13.0 (1.6-24.3)	183.6 (56.3-598.5)	5
8	105.6 (71.8-155.4)	86.9 (29.3-258.3)	17.6 (7.3-27.9)	411.7 (134.8-1257.1)	6
9 (O/N)	514.5 (508.5-520.5)	45.4 (13.4-126.1)	9.8 (4.4-15.1)	44.6 (16.1-123.6)	1

The table shows the different measurements made for each of the nine sampling periods. Geometric means are shown, except for the percentage of total quantity of allergen, which is an average. On each occasion, subjects collected 9 samples over 24 hours. This was performed only once by 11 subjects and one subject (5) collected a second sample on another day, shown as 5(2) , making a total of 13 occasions.

**Table 2:** Exposures in the 14 categories of activity as determined by the mixed model analysis of the nine samples collected over 24 hours by each subject.

Activity	n	Exposure		p
		pg/m^3^, GM (95% CI)	Ratio (95% CI)	
House, early morning	14	313 (118-832)	1.54 (0.57-4.14)	0.4
House, active in day	25	226 (102-495)	1.07 (0.47-2.45)	0.9
House, relax late evening	15	347 (134-896)	1.74 (0.67-4.58)	0.3
House, bedroom overnight	13	45 (17-117)	0.17 (0.065-0.45)	<0.001
Indoor, work, office	17	106 (43-262)	0.44 (0.17-1.11)	0.08
Indoor, work, laboratory	4	135 (19-960)	0.62 (0.09-4.49)	0.68
Indoor, social situation	8	273 (76-989)	1.30 (0.36-4.76)	0.7
Outside, social	14	163 (61-441)	0.74 (0.27-2.02)	0.5
Lunch, not at work	10	168 (53-527)	0.76 (0.24-2.43)	0.6
Travel, car	12	474 (163-1383)	2.43 (0.82-7.15)	0.1
Travel, public transport	7	1117 (289-4314)	5.80 (1.50-22.48)	0.01
Travel, walk, cycle	7	377 (92-1535)	1.82 (0.44-7.52)	0.4
Domestic cleaning	8	340 (89-1296)	1.64 (0.42-6.41)	0.5
House, bedroom, relax in day	4	673 (110-4112)	3.28 (0.53-20.16)	0.2

Results of the mixed-model analysis of the nine samples showing the exposures (pg/m^3)^ and the ratios of geometric mean exposure of that activity compared to geometric mean of all the other activities, for the 14 categories of activities. The total number of occurrences of each activity is shown as 'n'

## Discussion

This study strongly suggests mite aeroallergen exposure mainly occurs during the day, rather than at night, as previously thought. Personal exposure was frequently higher during active domestic and public activities and typically was lower in offices and in bed.

The consistency of exposure in some subjects and the high exposure in public suggests clothing may be an important source, as indicated by us for mite [11] and for cat allergen by others [12,13], although this does not discount the role of other domestic sources.

The average exposure at night may be low, despite the proximity to bed reservoirs, because people are largely immobile while asleep and so only infrequently re-aerosolise the large (>10 µm) particles [14–16] that carry most HDM allergen.

As daily aerosol exposure was not related to the allergen concentration in bed dust, the use of alternative proxies to the current convention of using the concentration of allergen in bed dust for establishing exposure should be explored.

The higher proportion of total HDM exposure occurring during the day may help explain why previous interventions, mainly directed at beds, have not yielded the anticipated clinical benefits [17].

There are several limitations. The difference in location of the IOM sampler on the shoulder during waking hours and on the bed head at night may have underestimated exposure in beds. While this was difficult to avoid given the practical logistics, these variations in location of between ~15 and ~40 cm of the IOM sampler to the nose may not consistently reflect what is inhaled. Also, the study only used 12 subjects, once, and so the findings should not be over-generalised, as they are likely to vary between days within subjects depending on their activities, as well as between subjects depending on many variables including age group, season, lifestyle, activity and country. Nonetheless the consistency of higher exposure during the day and early evening (Periods 1-8) compared to in bed is clear. These findings are also consistent with the recent study showing the high HDM aeroallergen exposure in classrooms, compared to lounge rooms, and where bedrooms had the lowest exposure, (derived from Figure 3 of [7]). They are additionally consistent with findings for another bioaerosol, endotoxin, where exposure depends a ‘personal cloud’ generated by activity [8,18] and with other studies of domestic particle exposure [15,19] also showing the importance of the type and intensity of physical activity. Further studies exploring these variables and the effect of small differences in the personal exposure zone are required.

In conclusion, this study suggests we currently have little idea when and where most HDM exposure occurs over time. Previous interventions directed at beds are unlikely to have greatly reduced total exposure. In order to test whether reducing domestic allergen exposure is of benefit to people with allergic asthma, the sources of individual aeroallergen exposure will need to be identified, and then interventions developed which are effective at reducing these. These concepts have been expanded on elsewhere [15,20].

## Supporting Information

Figure S1 Average personal exposure (pg/m^3^) for each of the 12 subjects. Each symbol represents the average exposure (pg/m^3^) for the nine different sampling periods over the 24 hours. Periods P1-P8 were of approximately 2 hours each, between 7am and 10:30 pm and P9 was overnight (~8 hrs). Subject 5 collected samples on 2 days in different locations during the day.
(TIFF)

Table S1 The 39 observed activities that were further summarised into 14 categories of activity. In total 39 activities were identified from a *post hoc* review of the diary records kept for each sample and from viewing the automated pictures taken by the iPod camera running Timelapse Pro worn by the subject. These were further summarised into 14 categories of similar activities for the mixed model analysis.
(DOC)

## References

[1] Sordillo JE, Webb T, Kwan D, Kamel J, Hoffman E et al. (2011) Allergen exposure modifies the relation of sensitization to fraction of exhaled nitric oxide levels in children at risk for allergy and asthma. J Allergy Clin Immunol 127: 1165-1172 plus U1441. doi:10.1016/j.jaci.2011.01.066. PubMed: 21463890. 10.1016/j.jaci.2011.01.066 PMC3137133 21463890

[2] Matsui EC, Sampson HA, Bahnson HT, Gruchalla RS, Pongracic JA et al. (2010) Allergen-specific IgE as a biomarker of exposure plus sensitization in inner-city adolescents with asthma. Allergy 65: 1414-1422. doi:10.1111/j.1398-9995.2010.02412.x. PubMed: 20560910. 20560910 10.1111/j.1398-9995.2010.02412.x PMC3345161

[3] Tovey E, Marks G (1999) Methods and effectiveness of environmental control. J Allergy Clin Immunol 103: 179-191. doi:10.1016/S0091-6749(99)70488-4. PubMed: 9949306. 9949306 10.1016/s0091-6749(99)70488-4

[4] O’Rourke SD, Tovey ER, O’Meara TJ (2002) Personal Exposure to Mite and Cat Allergens. J Allergy Clin Immunol 109: S47. doi:10.1016/S0091-6749(02)81227-1. 10.1067/mai.2000.110804 11080709

[5] Sakaguchi M, Inouye S, Yasueda H, Shida T (1992) Concentration of airborne mite allergens (*Der* I and *Der* II) during sleep. Allergy 47: 55-57. doi:10.1111/j.1398-9995.1992.tb02250.x. PubMed: 1590568. 1590568 10.1111/j.1398-9995.1992.tb02250.x

[6] Sakaguchi M, Inouye S, Yasueda H, Irie T, Yoshizawa S et al. (1989) Measurement of allergens associated with dust mite allergy. II. Concentrations of airborne mite allergens (Der I and Der II) in the house. Int Arch Allergy Immunol 90: 190-193. doi:10.1159/000235022. 10.1159/000235022 2583857

[7] Raja S, Xu Y, Ferro AR, Jaques PA, Hopke PK (2010) Resuspension of indoor aeroallergens and relationship to lung inflammation in asthmatic children. Environ Int 36: 8-14. doi:10.1016/j.envint.2009.09.001. PubMed: 19796820. 19796820 10.1016/j.envint.2009.09.001

[8] Delfino RJ, Staimer N, Tjoa T (2011) Personal endotoxin exposure in a panel study of school children with asthma. Environ Health 10: 69. doi:10.1186/1476-069X-10-69. PubMed: 21810249. 21810249 10.1186/1476-069X-10-69 PMC3161931

[9] Crisafulli D, Almqvist C, Marks G, Tovey E (2007) Seasonal trends in House Dust Mite allergen in children’s beds over a seven year period. Allergy 62: 1394-1400. doi:10.1111/j.1398-9995.2007.01533.x. PubMed: 17983374. 17983374 10.1111/j.1398-9995.2007.01533.x

[10] Glasgow NJ, Ponsonby AL, Kemp A, Tovey E, van Asperen P et al. (2011) Feather bedding and childhood asthma associated with house dust mite sensitisation: a randomised controlled trial. Arch Dis Child 96: 541-547. doi:10.1136/adc.2010.189696. PubMed: 21451166. 21451166 10.1136/adc.2010.189696 PMC3093241

[11] De Lucca SD, O’Meara TJ, Tovey ER (2000) Exposure to mite and cat allergens on a range of clothing items at home and the transfer of cat allergen in the workplace. J Allergy Clin Immunol 106: 874-879. doi:10.1067/mai.2000.110804. PubMed: 11080709. 11080709 10.1067/mai.2000.110804

[12] Patchett K, Lewis S, Crane J, Fitzharris P (1997) Cat allergen (Fel d 1) levels on school children’s clothing and in primary school classrooms in Wellington, New Zealand. J Allergy Clin Immunol 100: 755-759. doi:10.1016/S0091-6749(97)70269-0. PubMed: 9438482. 9438482 10.1016/s0091-6749(97)70269-0

[13] Karlsson AS, Andersson B, Renström A, Svedmyr J, Larsson K et al. (2004) Airborne cat allergen reduction in classrooms that use special school clothing or ban pet ownership. J Allergy Clin Immunol 113: 1172-1177. doi:10.1016/j.jaci.2003.12.590. PubMed: 15208601. 15208601 10.1016/j.jaci.2003.12.590

[14] Tovey ER, Chapman MD, Wells CW, Platts-Mills TA (1981) The distribution of dust mite allergen in the houses of patients with asthma. Am Rev Respir Dis 124: 630-635. PubMed: 7305119. 7305119 10.1164/arrd.1981.124.5.630

[15] Tovey E, Ferro A (2012) Time for New Methods for Avoidance of House Dust Mite and Other Allergens. Curr Allergy Asthma Rep 12: 465-477. doi:10.1007/s11882-012-0285-0. PubMed: 22833251. 22833251 10.1007/s11882-012-0285-0

[16] Gore R, Boyle RJ, Hanna H, Custovic A, Gore C et al. (2011) Personal allergen exposure is increased by turning over in bed and improved by temperature-controlled laminar airflow. Clin Exp Allergy 41: 1848-1848.

[17] Gøtzsche PC, Johansen HK (2008) House dust mite control measures for asthma: systematic review. Allergy 63: 646-659. doi:10.1111/j.1398-9995.2008.01690.x. PubMed: 18445182. 18445182 10.1111/j.1398-9995.2008.01690.x

[18] Rabinovitch N, Liu AH, Zhang LN, Rodes CE, Foarde K et al. (2005) Importance of the personal endotoxin cloud in school-age children with asthma. J Allergy Clin Immunol 116: 1053-1057. doi:10.1016/j.jaci.2005.08.045. PubMed: 16275375. 16275375 10.1016/j.jaci.2005.08.045

[19] Ferro AR, Kopperud RJ, Hildemann LM (2004) Elevated personal exposure to particulate matter from human activities in a residence. J Exp Anal Environ Epi 14: S34-S40. doi:10.1038/sj.jea.7500356. 10.1038/sj.jea.7500356 15118743

[20] Tovey ER, Marks GB (2011) It’s time to rethink mite allergen avoidance. J Allergy Clin Immunol 128: 723-U370. doi:10.1016/j.jaci.2011.07.009. PubMed: 21855978. 21855978 10.1016/j.jaci.2011.07.009

