# Supplementary figures and images for: Most Personal Exposure to House Dust Mite Aeroallergen Occurs during the Day
Source: PLoS One. 2013 Jul 24;8(7):e69900. doi: 10.1371/journal.pone.0069900 (PMC3722239; doi:10.1371/journal.pone.0069900)

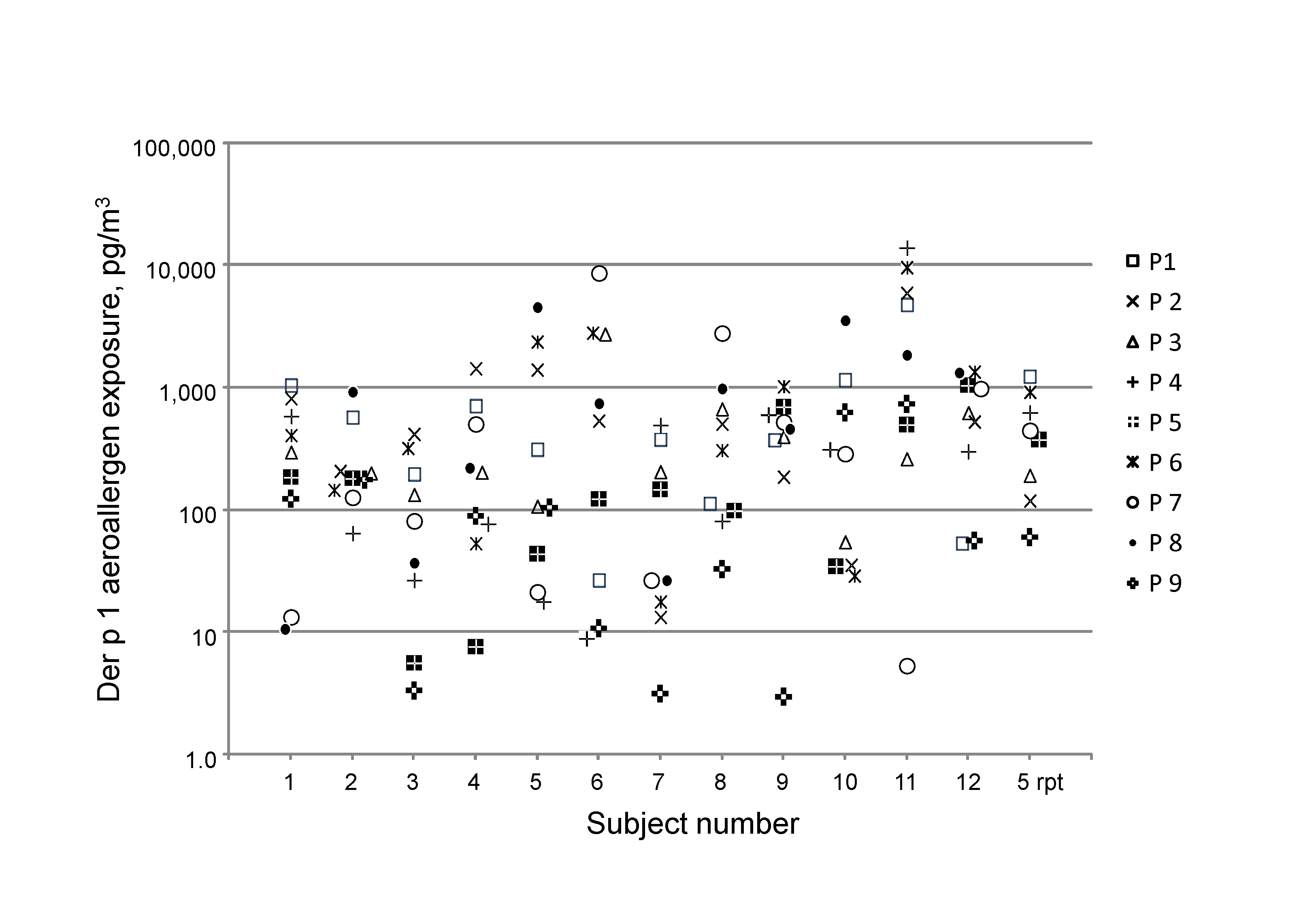

Supplement: Figure S1 — Each symbol represents the average exposure (pg/m3) for the nine different sampling periods over the 24 hours. Periods P1-P8 were of approximately 2 hours each, between 7am and 10:30 pm and P9 was overnight (~8 hrs). Subject 5 collected samples on 2 days in different locations during the day. (TIFF) [file pone.0069900.s001.tiff]
