# Supplementary material for: Most Personal Exposure to House Dust Mite Aeroallergen Occurs during the Day
Source: PLoS One. 2013 Jul 24;8(7):e69900. doi: 10.1371/journal.pone.0069900 (PMC3722239; doi:10.1371/journal.pone.0069900)
Supplement: Table S1 — In total 39 activities were identified from a post hoc review of the diary records kept for each sample and from viewing the automated pictures taken by the iPod camera running Timelapse Pro worn by the subject. These were further summarised into 14 categories of similar activities for the mixed model analysis. (DOC) [file pone.0069900.s002.doc]

**Table SI.** **The 39 observed activities that were summarised into 14 categories of activity**

| 1. House early morning |
| --- |
| a. In bathroom |
| b. Dressing |
| c. Breakfast |
| d. Making bed |
| e. Preparation for leaving for work |
|  |
| 2. At home and active in the day or early evening |
| a. Studying |
| b. Working on computers |
| c. Organising, watching TV or reading in the day |
| d. Preparing meals in the kitchen |
|  |
| 3. At home and relax after evening meal |
| a. Watching TV |
| b. Reading |
| c. Playing games |
| d. Social talking, eating, drinking |
| e. Preparation for bed |
|  |
| 4. Relax during day in bedroom’ (returned to bed after breakfast) |
| a. Snacking |
| b. Use of computer |
| c. Watching TV on bed |
|  |
| 5. In bed at night, sleep (from getting into bed until getting up in morning) |
|  |
| 6. Indoors in office |
|  |
| 7. Indoors in laboratory |
|  |
| 8. Indoor social activity away from home |
| a. Bar |
| b. Yoga |
| c. Gym |
| d. Shopping |
| e. Restaurant |
|  |
| 9. Outside (social context or relaxing) |
| a. At the beach |
| b. Relaxing on the balcony |
| c. Playing with dogs |
| d. Working at an event |
|  |
| 10. Lunch (typically away from work) |
|  |
| 11. Travel by car |
|  |
| 12. Travel by public transport |
| a. Train |
| b. Bus |
| c. Ferry |
|  |
| 13. Travel / outdoor |
| a. Walk |
| b. Cycle |
|  |
| 14. Cleaning |
| a. Sweeping |
| b. Vacuuming |
| c. Laundry |
